# A 12-Month Randomized Controlled Trial to Assess the Impact of Telemedicine on Patient Experience and Care Continuity

**DOI:** 10.7759/cureus.53201

**Published:** 2024-01-29

**Authors:** Ruchi Garg, Akshi Walecha, Vinay Goyal, Aditi Mehra, Mayank Badkur, Ravi Gaur, Indra Singh Choudhary, Yatin Talwar

**Affiliations:** 1 Hospital Administration, All India Institute of Medical Sciences, Jodhpur, IND; 2 Anesthesia, All India Institute of Medical Sciences, Jodhpur, IND; 3 Physical Medicine and Rehabilitation, All India Institute of Physical Medicine and Rehabilitation, Mumbai, IND; 4 Hospital Administration, Government Medical College & Hospital, Chandigarh, IND; 5 General Surgery, All India Institute of Medical Sciences, Jodhpur, IND; 6 Physical Medicine and Rehabilitation, All India Institute of Medical Sciences, Jodhpur, IND

**Keywords:** statistics & numerical data, economics, telemedicine, health services, health care, outcome assessment, health services research

## Abstract

Background: Telemedicine is the use of electronic information to communicate technologies to provide and support healthcare when distance separates the participants. Satisfaction and engagement of patients are key resource indicators for any healthcare setup and healthcare provider for evolving the care continuum (a system that provides a comprehensive range of health services so that care can evolve with the patient over time) and ensuring continuous quality improvement in the systems. As the latest remarkable strategy to connect with patients for consultations and follow-up, telemedicine has been of pivotal importance, especially during the coronavirus disease 2019 (COVID-19), where medicinal services utilize digital sound, video, and information interchanges to remotely access and provide care.

Materials and methods: A cross-sectional study was planned during the second wave of the COVID-19 pandemic from April 2021 to April 2022 to assess the impact of telemedicine in essential healthcare delivery by super specialty tertiary care healthcare setup, which is also a medical college, by three consultants and a physiotherapist.

Results: There was a significant improvement in satisfaction scores and an improvement in the approach of patients towards telemedicine was observed. Various other parameters, like readmission compliance with medications and a reduction in ED times, were also observed. Finally, clinical endpoints were captured, and the correlation between readmission and medicine adherence was found to be strongly correlated (r = 0.9). A p-value of the reduction in utilization times of ED (emergency department), readmission, and medicine adherence was found to be highly significant

Conclusions: Telemedicine is the need of the hour and is now an essential part of healthcare. Its acceptance post-COVID-19 pandemic and adaptability into existing healthcare setups would deliver fruitful results.

## Introduction

Telemedicine is a clinical application that connects a patient or healthcare seeker with a healthcare provider by means of digital, social media, or a technology platform. It is also known as distance healing, which potentially helps patients with self-management [1]. Telemedicine is the utilization of clinical information by means of electronic or digital media to improve a patient’s communication status and well-being. The World Health Organization (WHO) characterizes telemedicine as "the conveyance of social insurance administrations, where separation is a factor, by all human services experts utilizing information and communication innovations for the trading of substantial information for finding, treatment, and avoidance of infection, what's more, wounds, research, and evaluation, and for the proceeding with instruction of social insurance suppliers, all in light of a legitimate concern for propelling the well-being of people and their networks" [1,2]. The Institute of Medicine (IoM) characterizes telemedicine as "the utilization of electronic information and communications innovations to give and bolster social insurance when separation distance is greater" [2].

It is estimated that a great number of patients with illnesses adapted to teleconsultations during the coronavirus disease 2019 (COVID-19) pandemic. The effect was more profound during the second and third waves, which continued from April 2021 to April 2022 in India, when the number of patients seeking teleconsultations also increased because of micro-containment zones, lockdowns, and enforcement of travel restrictions, besides news of hospitals stopping regular outpatient departments or long waiting times at the consultation chambers. The adaptability of telemedicine during the second wave and its ease of use probably led to patients preferring this mode of consultation compared to face-to-face consultations. Besides, when the pandemic was at its highest level, there was also an unmet need for patients with chronic diseases or those on immunosuppressants to ensure their safety, in addition to seeking an appointment for consultation with the care provider to ensure the care continuum. This paved the way for the adaptation of teleconsultation or telemedicine in the Indian health system.

Telemedicine is currently utilized in an array of healthcare settings, and despite the fact that there are a number of concerns associated with this innovation, its usage has the ability to provide a few solutions to the projected issues of care management. While telemedicine may or may not drive further advances, it will almost certainly impact the traditional method of managing care. While clearly, business is influencing the practice of healthcare, possibly an added business perspective might improve the situation by focusing on the elements of the common sense of telemedicine as a suitable approach for medicinal service conveyance [3].

The medical services profession is undergoing tremendous change, and many hospitals and administrators are attempting to be updated and adapted to the new modalities. It should also be noted that when a new technology or modality is introduced, the adaptations of both the healthcare practitioner and the healthcare seeker vary, as do the satisfaction indices. As a result, this study was designed with a few objectives in mind. The goals were to analyze the influence of telemedicine on vital health care delivery, the adaptability of health care practitioners to use teleconsultations, and user adaptation and satisfaction with teleconsultation. With the prospect of greater health expenditure, along with the ongoing COVID-19 epidemic, India's healthcare market situation has drastically transformed. Hospitals, public health, medical gadgets, telehealth, the pharmaceutical industry, medical tourism, health insurance, clinical trials, and so on are all part of the healthcare sector all are attempting telemedicine and thus increasing the usability and possibility of further growth in this sector. Global insights into the digital health market of the healthcare sector too forecast a compound annual growth rate (CAGR) of 28.5% by 2026, as digital transformation is adopted. While the CAGR in the telehealthcare category is expected to be 26.2% by 2026 [1,3].

The expanding number of COVID-19 cases around the globe prompted inconveniences of lockdowns and severe social removal measures by legislatures around the world. The profoundly infectious nature of the infection had driven the guardians to utilize computerized well-being advancements, for example, telehealth to effectively treat patients. Doctors resorted to the internet and mobile device-based healthcare services to counsel and monitor patients in fear of cross-infection and community transmission. This has driven upon to push the market development. As an expanding number of patients go to advanced well-being advances, organizations were seen extending their abilities to manage the abrupt increment in the understanding volume, which will eventually help in the bigger and better market demand for telemedicine. The background of the study was based on 5 challenges faced by healthcare institutions during the COVID-19 pandemic: (1) Overutilization of emergency departments during the pandemic and measures to control them; (2) Increased point of care and risk of readmission in cases where checks on post-discharges follow-up were not monitored; (3) Out of pocket expenses for visiting a healthcare setup for consultations; (4) Universal affordable accessible healthcare to all; and (5) Patient privacy [4,5].

It is unlikely that telehealth alone can reverse disease pathology or predictable outcomes of the disease despite the above, the majority of published telehealth studies have focused on patient populations selected by diagnosis, such as cancer patients / Chronic heart, renal, or liver failures [5,6].

This research was designed with the goal of establishing an early diagnosis, directing patients to safe home care while controlling comorbidities and limiting viral transmission, further researching satisfaction scores, improving scores on the use of multiple teleconsultations, and measuring end points of telehealth.

## Materials and methods

A cross-sectional study was planned during the second wave of the COVID-19 pandemic from April 2021 to April 2022 to assess the impact of telemedicine in essential healthcare delivery by a super specialty tertiary care healthcare setup, which is also a medical college [7]. The study was conducted by three physicians (a general medical practitioner, a pulmonologist, and a psychiatrist), and a physiotherapist. This team was entrusted with the responsibility of providing tele-consultations to all confirmed or suspected COVID-19 patients or those suffering from post-COVID infection syndrome. The consent for teleconsultations, privacy, and consent for the study were obtained from the patients, and following recovery, a validated, pre-tested questionnaire was forwarded to them for registration of their responses. The validity and reliability of the questions selected or designed in the questionnaire used for the study were validated as per the Telemedicine Satisfaction Questionnaire [2]. The questionnaire was based on the format set by the HTF 402 National First Nations Telemedicine Research Project, 2014 [2]. The questions were based on educational background, ease of achieving a consultation, and personal interview questions about patient appointments, home care, readmissions, perceived advantages, disadvantages, and barriers to telemedicine for the study subjects attending telemedicine medicine from April 2021 to April 2022. The methods and study design were adherent to the CONSORT Statement Guidelines. Guidelines as per the Declaration of Helsinki and good clinical care guidelines were also followed in letter and spirit. All patients attended during the peak of the second and third wave of COVID-19 from April 2021 to April 2022. All patients given consultations during these 365 days were asked for consent to be included in the study, and those who provided consent were included in the study. The study was double-blinded and consisted of open questions to exclude bias. Further, the data entry randomized the responses based on a lottery system and coded the responses to ensure randomization and reduce potential bias.

The team provided an overall 7578 tele-consultations to COVID-19-positive patients and to patients who were suffering from post-COVID infection syndrome. Out of these 7578 patients, 1253 were found to be serious enough to be treated on a home-care basis and were advised admission to inpatient or intensive care units. 19 patients refused to be a part of the study. A total of 6306 patients were included as the sample size in the study. Inclusion criteria included all new patients attending the telemedicine unit, and exclusion criteria included patients who didn’t give consent, patients who didn’t complete the teleconsultations, sick patients, and psychiatric and pediatric patients.

The aim of the study was explained to all of the study participants, and their consent was obtained before the form-filling and personalized interview. Also, as per good clinical practices, the strict confidentiality of the information was maintained, and calling was performed under supervision. Ethical clearance was also taken from the ethics committee of the institute where the team worked. All of the patients or teleconsultation seekers were also informed of the Telemedicine Practice Guideline of 2020 by the Government of India and the legal implication of the same in detail prior to the consultation as a rule.

A semi-structured questionnaire including medical records, and patient prescriptions along with a validated pretested questionnaire (Telemedicine Satisfaction Questionnaire) to assess the satisfaction, benefits, and barriers to telemedicine was used [2-4]. The information collected was converted into a computer-based spreadsheet using the Statistical Package for Social Sciences (SPSS), version 21.0 (IBM Corp., Armonk, NY) software. Descriptive statistics such as mean standard deviation and percentages were used to describe the data collected in the present study. Statistical analysis was done using the chi-square test wherever applicable.

## Results

A total of 6306 patients were surveyed from April 2021 to April 2022. Out of the total respondents, approximately 52%, i.e., 3321 patients were females, and the remaining 2985 were males, which is approximately 48% of the sample size. Of the female respondents, 2233 were of mean age of 36.18 (±11.26) years, with a range of 21-39 years. Only 85% of the respondents were literate, with males exceeding females by over 10 percent. Around 30% were unemployed or homemakers, and 30% were on a work-from-home (WFH) basis.** **

It was observed that out of 6306 subjects, 5990 had completed high school and 5612 had completed college. Among the patients, 3740 (59%) had a previous history of illness, comorbidity, or disability. The baseline demographics of 6303 patients have been compiled and tabulated in Table 1.

**Table 1:** Demonstrating the basic demographics of subjects

Basic demographics	N = 6306 telemedicine
Age mean (SD)	36.18 (+/- 11.26 years)
Sex	3321 females (52%)
Religion	Hindus 84% (5297)
Education, n (%)	
Completed school	95% (5990)
Completed college	89% (5612)
Employed n (%)	30% unemployed; 30% work from home; 40% working
Sick/disability/chronically Ill	59% (3740)
Number of diagnoses, with complications	COVID-19 62%; musculoskeletal pathology 5%; lungs and heart pathology 19%; neuropsychiatry pathology 3%; psychiatry pathology 9%; miscellaneous 2%
Median number of follow-up appointments on discharge	3

Since the telemedicine and telehealth projects were primarily designed to reduce the ED (emergency department) load and reduce the turnaround times, COVID-19-suspected and COVID-19-positive patients were offered consultations. Median 3 follow-ups were given to the patients who were offered telehealth. The patients discharged from the hospital were also provided follow-up. The observers found that 62% of the patients were COVID-19-positive and were managed by the team at home, with the help of telemedicine and consultations. The 1253 patients who were found to be serious and were advised admission to inpatient/intensive care units are already excluded from the study. Pre and post-consultation scores of the patients were elicited and complied, as shown in Table 2.

**Table 2:** Pre- and post-consultation scores of the study

Query	Pre-consultation score	Post-consultation 1 score	Post-consultation 2 score	Post-consultation 3 score
How would you rate your health? n (% Good/Very Good) (0 = Good/Very Good, 1 = Poor/Fair)	50%	65	65	68
Access to care	90%	95	99	99
Confidence in health management (0–10: Least Confident-Most Confident, respectively) Median	9(4,10)	9 (4,10)	9.2(5,10)	9.5(5,10)
Computer/tech savviness: do you use a computer on a regular basis?	82%	82%	82%	83%
How comfortable are you with using technology like a smartphone or tablet? (0–10: Least Comfortable-Most Comfortable, respectively) Median (Range)	6 (0,10)	7 (0,10)	7.2 (1,10)	8(4,10)
Do you own a cell phone?	99%	99%	99%	99%
Do you have internet service in your home?	99%	99%	99%	99%
Do you have difficulties with your cell service, whereby you experience dropped calls or poor reception?	4%	4%	4%	4%
How confident are you that telehealth may help your healthcare? (0–10: Least Confident-Most Confident, respectively) Median	6(0,10)	7 (0, 10)	7 (0, 10)	8(0,10)

Overall it was also observed that 53% of the study subjects had not previously heard of telemedicine, especially during the first wave of COVID-19. This can be attributed to the literacy levels, rural background, and the older age of patients who were not technologically advanced. However, because of the orientation and education of the masses by the government, media houses, and hospitals, it was observed that many knew of telemedicine/telehealth during the COVID-19 second wave. 

Twenty percent of these patients who were chronically ill or had a chronic disease were oriented about telemedicine by their treating doctors or fellow patients. More than 45 percent of the patients were oriented and informed about the teleconsultation focused on COVID-19 from social media and information, education, and communication (IEC) fliers/brochures at hospitals. Since the medical establishment had started teleconsultation focused on COVID-19 after the first wave of the pandemic, where a decision was taken by the medical advisory board to reduce the workload of the outpatient department and to provide affordable telehealth to society, the number of patients seeking telemedicine jumped constantly over a week of announcements. The jump in patients can also be attributed to the SMS sent by the information technology team of the hospital to all registered patients; since the hospital and college was one of the oldest in the city, the bias of reaching out to a huge number of patients, however, cannot be ruled out.

**Figure 1:**
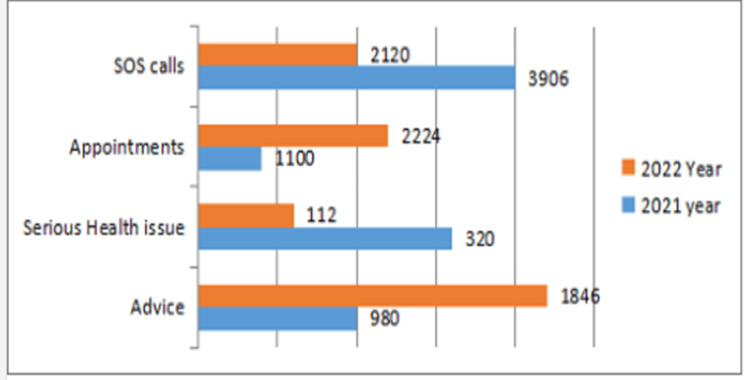
Demonstrating the pattern of calls received by the telemedicine team February 2021 to July 2021 is taken as year 2021, and calls received from August 2021 to February 2022 have been taken in year 2022

It was noticed that the SOS calls received for the year 2021 were replaced by almost 50% in the year 2022 and there was an increase of more than 50% in calls seeking advice. The calls for seeking appointment of clinicians or emergency beds also saw a rise. This phenomenon led to the starting up of a dedicated CRM (customer relationship management) center in the telemedicine department. The calling patterns of patients are shown in Figure 1.

During the interval of the study, it was observed that out of the sample size, more than 95 percent of patients advised medications and physiotherapy recovered with only 4.5 percent of patients needing admission. Also, more than 91% of the patients took telemedicine consultation more than 3 times for a single episode of illness, and out of 91%, not even 1% (significant p > 0.005) visited the hospital. It was also observed that there was a continuous rise in telemedicine calls and referrals since July 2021. When the researchers sub-analyzed the clinical data, it revealed that only 22.1% of the telemedicine consultations influenced the making of a definite diagnosis, and nearly 78.3% of consultations contributed to clinical management and treatment. Also, it was found that during the pandemic, the most common reason for willingness to use telemedicine was the availability of a specialist consultation followed by counseling and the second most common reason was easy availability of a second opinion. The commonest barrier to using telemedicine observed was that the patient felt that he/ she was not in direct contact with the doctor or the consultant. Of those surveyed, only 32% were satisfied by the telemedicine consultation in the first instance which increased to 65% by the next year. 

**Figure 2:**
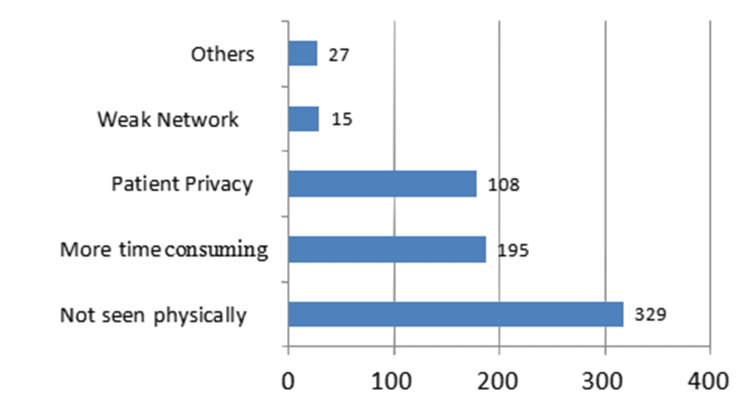
Figure representing barriers to the use of telemedicine among the study subjects

The major barrier found during the study was not being physically seen by the consultant. Around 329 (5.2%) patients were dissatisfied because the healthcare provider was not able to physically inspect them, 195 (3.09%) patients found it more time-consuming, and 108 (1.71%) patients were concerned with privacy as they felt it uncomfortable to discuss health issues in front of family members, less than 0.1 percent patients had network issues. Barriers to telemedicine are elicited in Figure 2.

The clinical endpoints of the telemedicine services have been summarized as follows: where it was found that ED utilization was significantly reduced and medication adherence was significantly increased, there was a slight positive correlation between the two. The correlation was found to be positive and moderate (r=0.20). The correlation between readmission and medicine adherence was found strongly correlated (r=0.9). The p-value of the reduction in utilization times of ED, readmission, and medicine adherence was found highly significant. These have been compiled in Table 3 depicting the clinical endpoints of telemedicine.

**Table 3:** Clinical endpoints of telehealth *p<0.05 statistically significant; **p<0.001 statistically highly significant; Student's t-test was used to test the significance ED: Emergency department

Effect	Point estimate	p-value	Coefficient of determination
ED utilization	90% reduced to 55%	0.000714*	0.036
Readmission	4.5% from 7.8 %	<0.00001*	0.058
Medication adherence	98%	0.000781*	0.002
Follow up visit	Reduced by 55%	0.011*	0.2
Death	Nil	Nil	Nil

The Kaiser-Meyer-Olkin measure (0.90) indicated strong relationships between the variables. With data from patients, factor analysis was conducted using the principal components analysis extraction method with varimax rotation on the satisfaction studies. Bartlett’s Test of Sphericity was significant (<0.001), providing support for an appropriate procedure with these data. 

## Discussion

Telemedicine emerged as a crucial tool during the COVID-19 pandemic, playing a pivotal role in both managing the pandemic and delivering healthcare in a safer and more efficient manner. The outcomes of the research were quite similar to the other studies published during the pandemic. The notable interpretations from the study included the following: (1) Increased access to healthcare: Telemedicine provided invaluable access to healthcare for patients, especially those at high risk for severe COVID-19 complications, by enabling them to receive consultations and follow-up care remotely. This minimized unnecessary exposure to the virus and allowed for timely management of symptoms and ongoing care. Nearly all research conducted during the pandemic supports this evidence [6-9]; (2) Reduced burden on healthcare systems: By facilitating virtual consultations and remote monitoring, telemedicine took center stage by helping optimize healthcare resources, alleviating the strain on overburdened healthcare systems during the pandemic peak. This allowed for better allocation of resources to those in critical need. The utility of telemedicine in healthcare, especially during pandemics, has also been studied and mentioned in research by Colbert et al. and Ahmed et al. [6,9]; (3) Improved safety for patients and healthcare workers: Telemedicine minimized the risk of exposure to COVID-19 for both patients and healthcare workers by reducing unnecessary in-person visits. This prevented potential transmission and outbreaks, promoting safer healthcare delivery. telemedicine contributed to managing COVID-19 patients while maintaining social distancing and promoting patient safety; this has also been observed by Colbert et al. [6,7]; (4) Enhanced patient convenience and satisfaction: Telemedicine offered substantial convenience for patients, allowing them to access healthcare from the comfort and safety of their homes. This contributed to increased patient satisfaction and greater engagement with health services.

The crucial role telemedicine played in managing the COVID-19 pandemic and its potential for improving healthcare delivery in the future cannot be ignored.

Telemedicine has been prevalent in Indian healthcare for a decade; however, after the launch of the Telemedicine Act and the COVID-19 pandemic, it has found acceptance among the masses. During the study and upon discussion with patients and care providers, it was observed that there are some benefits as well as blockages and disadvantages. When compared to other studies, it was observed that the majority of participants in other studies were younger and were from the age group of 18-39 years. This may be a confounding factor, as elders and those over 60 may face more difficulty adapting to telemedicine. In our study,it was found that only 47% of the subjects had previously heard of telemedicine, which was very unlikely in developing countries.

Most probably, this trend may also be linked to the literacy level among the study subjects and the age of the patients. Further hesitancy to use telemedicine due to the barriers elicited in the study may be a reason for the low numbers earlier. In about 39.1%, the telemedicine consultation influenced making a definite diagnosis, and in nearly 58.3%, the consultation contributed to clinical management. The reason for the acceptance of telemedicine was the ongoing pandemic. Not being in direct contact with a doctor came out to be the commonest barrier which was similar to the findings of Apollo Tele Health Services. Of those surveyed, 80% were satisfied with the telemedicine consultation in contrast to the findings of Acharya and Rai [5,9,10].

This might be due to differences in socio-economic background, education of samples, counseling done by the team, and reputation of the hospital and college along with the telemedicine consultations. There is a need to further escalate the scope and services of telemedicine consultations to all patients, where all people from all forms of life can be catered to telemedicine consultations.

## Conclusions

Telemedicine is a boon to the fields of medicine and healthcare. Our experience demonstrates that besides offering consultation, it can also be used for various other hospital operations, like bed allocation and distribution. The most common reasons for willingness to use telemedicine were specialist consultations. For 39.1% of the study sample, the telemedicine consultation influenced making a definite diagnosis; for 58.3% of the study population, the consultation contributed to clinical management. Changes should be made to decrease the waiting time. Secondly, the doctor’s perspective should also be considered. Awareness sessions should be planned beforehand for the consultation, especially when the patient needs to talk to the consultant in privacy.
